# Linear atrophoderma of Moulin: A rare case report and review of the literature

**DOI:** 10.1002/ski2.424

**Published:** 2024-07-21

**Authors:** Moatasem Hussein Al‐janabi, Sdrah Diab, Ghina Aljammal, Lina Kassab, Zuheir Al‐Shehabi, Lina Al‐Soiufi

**Affiliations:** ^1^ Department of Pathology Cancer Research Center Tishreen University Hospital Lattakia Syria; ^2^ Department of Dermatology and Sexually Transmitted Disease Tishreen University Hospital Lattakia Syria; ^3^ Faculty of Medicine in Damascus University Damascus Syria; ^4^ Department of Dermatology National Hospital Lattakia Syria

## Abstract

Linear atrophoderma of Moulin (LAM) is an uncommon dermatological disease characterised by linear, depressed plaques typically following Blaschko's lines. LAM generally occurs in adolescence or early adulthood and is more commonly observed in females. The aetiology of LAM is still unclear. We report a rare case of LAM in an 18‐year‐old female presenting with an atypical Blaschkoid distribution (multiple band‐like pattern) on her right trunk. A clinical examination and histopathological analysis were performed to make the diagnosis. Partial improvement was obtained with calcipotriol and colchicine. LAM is a rare condition; we were only able to identify 23 case reports in the published literature. The findings of this report contribute to the limited literature on LAM, highlighting the clinical variability of LAM and suggesting potential novel variants beyond the classic presentation, emphasising the importance of recognising diverse manifestations for accurate diagnosis and management. Early recognition of LAM is crucial for appropriate treatment and improved patient outcomes. Further research is needed to elucidate LAM's aetiology and underlying mechanisms to facilitate the development of more targeted therapeutic strategies.

## Graphical abstract

Linear atrophoderma of Moulin (LAM) is an uncommon dermatological disease characterised by linear, depressed plaques on the skin. TLAM is a rare condition; we were only able to identify 23 case reports in the published literature. The findings of this report contribute to the limited literature on LAM, highlighting the importance of accurate diagnosis and management.

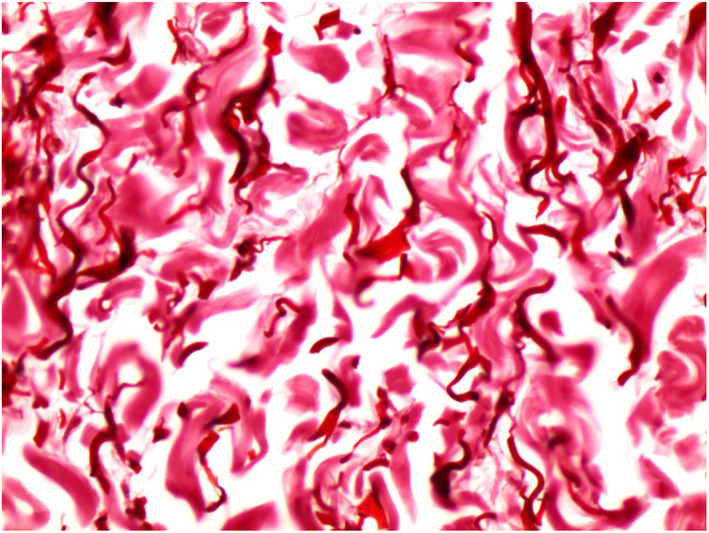

## 1 INTRODUCTION

Linear atrophoderma of Moulin (LAM) is a rare dermatological disorder first described by Moulin et al. in 1992.^1^ It presents as asymptomatic hyperpigmented atrophic bands‐like lesions primarily found on the trunk and follows Blaschko's lines.^2^ LAM typically appears in early adolescence or childhood.^3^ The aetiology of LAM remains unclear, and its pathogenesis is not well understood.^4^ Histologic findings demonstrate hyperpigmentation of the basal cells along with mild thickening of the collagen fibres in the dermis and a sparse perivascular lymphocytic infiltrate.^5^ There is no standardized treatment for LAM. Some trial therapies, such as topical calcipotriol, aminobenzoate or systemic methotrexate, and intralesional platelet‐rich plasma therapy, demonstrated a partial response.^6^ We present a unique case of LAM with an atypical Blaschkoid distribution, prompting consideration of a new variant of the disease. Understanding such variations is crucial for accurate diagnosis and effective management.

## 2 CASE PRESENTATION

**FIGURE 1:**
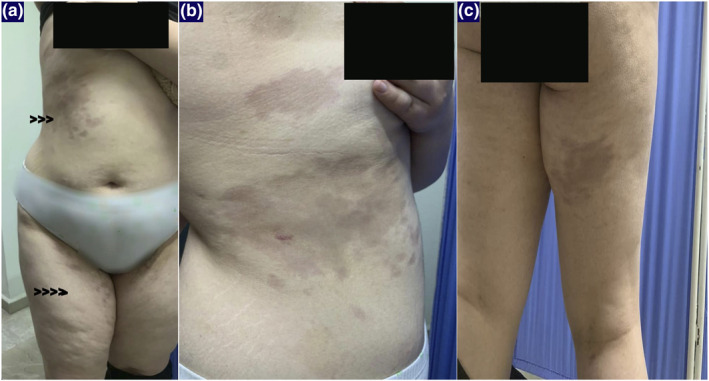
A clinical images of the patient show linear hyperpigmented atrophic patches with an atypical Blaschkoid distribution (multiple band‐like pattern) on the right trunk (a and b), extending along Blaschko's lines to the right buttock and thigh (c).

**FIGURE 2:**
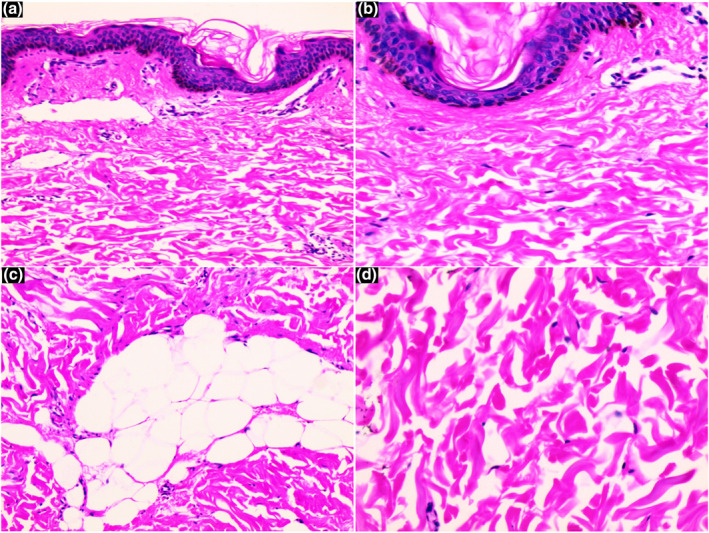
H&E‐stained sections of the lesion. Panels (a–d) show normal epidermis with mild linear hyperpigmentation of the basal layer, altered collagen fibres, fragmentation of elastic fibres, spare perivascular lymphocytic infiltrate, and thinning of the subcutaneous layer (40×, 100×, 400×, 400×).

**FIGURE 3:**
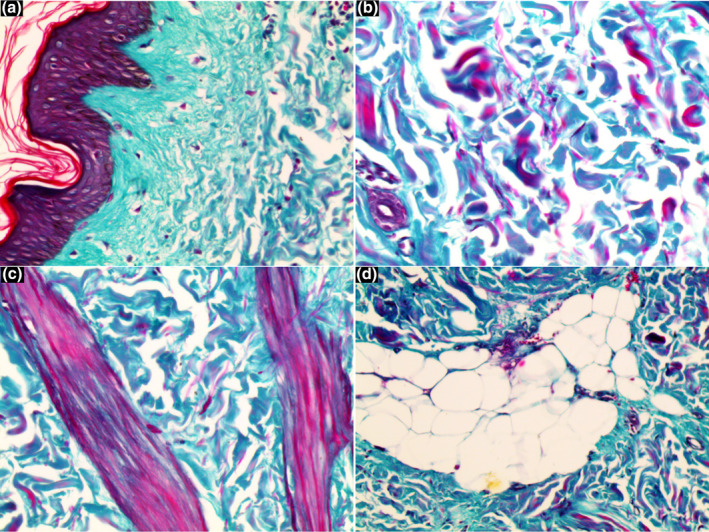
(a–d) Masson‐trichrome stain section revealed altered collagen fibers. (a) Shows a section of skin with the epidermis on the left side and the underlying dermis, indicating a clear boundary and altered collagen fiber arrangement in the dermal layer. (b) Displays disorganized and loosely arranged collagen fibers with a wavy pattern and varying thickness. (c) Highlights densely packed and thickened collagen fibers in a more organized manner, suggesting regions of increased collagen deposition. (d) Illustrates a section with both collagen fibers and adipose tissue, showing the interaction between the altered collagen matrix and fat cells.

**FIGURE 4:**
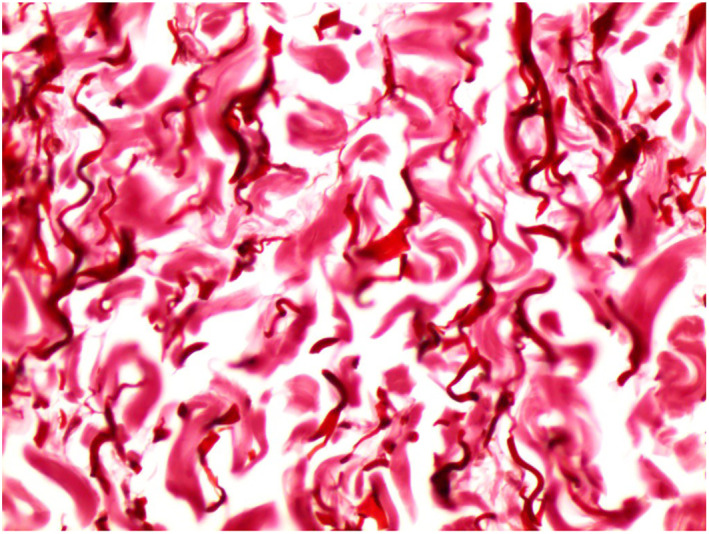
Orcein stain section revealed fragmentation of elastic fibres.

An 18‐year‐old girl presented with a history of asymptomatic unilateral, light brown, atrophic plaques affecting the right side of her trunk and right thigh. No significant medical or family history was found. Physical examination showed linear hyperpigmented atrophic plaques on the right trunk (Figure 1a) extending down to the right buttock and right thigh following Blaschko's line involvement (Figure 1b). Notably, the lesions displayed an atypical Blaschkoid distribution (multiple band‐like pattern), which is uncommon in typical cases of LAM. No signs of inflammation or sclerosis on the lesion were noted. Laboratory investigations, including CBC, ESR, liver, and renal function were within normal limits. A skin biopsy from a linear hyperpigmented plaque was performed. Histopathology of the lesion revealed a normal epidermis with mild linear hyperpigmentation of the basal layer, altered collagen fibres, fragmentation of elastic fibres, sparse perivascular lymphocytic infiltrate, and thinning of the subcutaneous layer (Figure 2). Masson‐trichrome and orcein stain sections revealed altered collagen fibres (Figure 3) and fragmentation of elastic fibres (Figure 4). Our primary consideration for the differential diagnosis was the atrophoderma of Pasini and Pierini (APP). While both conditions display similar features of atrophy and hyperpigmentation, it's important to note that APP typically does not conform to Blaschko's lines. Additionally, we considered linear morphea, distinguished by the absence of sclerosis and inflammation. The LAM was diagnosed based on clinical and histopathological features. The patient was treated with calcipotriol cream, colchicine tablets 0.5 mg per day, and vitamin E tablets for 3 months. We observed partial improvement in pigmentation during the early stage.

## 3 DISCUSSION

LAM is a rare clinical entity that usually manifests in childhood or early adolescence.^3^ In the medical literature, there have been reports of 23 cases of LAM to date (Table 1), making this condition extremely rare.^6^ The disease often presents asymptomatic hyperpigmented atrophic linear bands along Blaschko's lines without prior inflammation or sclerotic appearance. Later literature reported atypical LAM cases manifested as telangiectasias, erythematous plaques, dissemination to the trunk and extremities, and bilateral distribution along Blaschko's lines.^1^, ^2^, ^20^ Our case notably exhibited an atypical Blaschkoid distribution (multiple band‐like pattern), which is uncommon in typical cases of LAM. This unique presentation prompts consideration that our case may represent a novel variant of the LAM. Aetiology remains vague; it was suggested that early somatic mutation, resulting in genotypic and phenotypic mosaicism, might be the reason for Blaschko's line involvement.^21^ Lopez et al. suggested that the prevalence of LAM might be overestimated since histological findings of some reported cases might be compatible with other clinical conditions.^21^ Differential diagnoses of LAM include dermopathy following Blaschko's lines, such as incontinentia pigmenti, linear and whorled naevoid hypomelanosis, lichen striatus, and epidermal nevi. In addition, it is essential to differentiate LAM from the APP, which also presents with similar atrophy and hyperpigmentation but does not follow Blaschko lines.^15^ Differentiation between the LAM and morphea remains a challenge. However, LAM differs from linear morphea in the absence of sclerosis and inflammation.^6^ There are various clinical and histologic similarities between LAM, APP, and morphea. Thus, some of the literature suggests that LAM may not be a distinct entity and may be a Blaschko‐linear variant of APP, and APP would be considered an abortive form of morphea.^6^ Histopathological features that characterise LAM are hyperpigmentation of the basal cells, slight thickening of the dermis's collagen fibres, and sparse perivascular lymphocytic infiltration.^5^ The latter two are the most common findings in LAM.^21^ Acanthosis, epidermal atrophy, and decreased or fragmented elastic tissue might be found. Dermal atrophy signs are not detectable. The atrophic appearance on the clinical examination might be attributed to a reduction in subcutaneous tissue, as demonstrated by ultrasound imaging.^17^ No standard treatment regimen for LAM has been developed yet. Most treatments typically fail to effectively treat the LAM lesion. However, some papers reported partial improvement with intravenous penicillin together with topical PUVA therapy, oral Potaba (potassium para‐aminobenzoate), high‐dose vitamin E (ve 400 IU/day), topical clobetasol propionate, and systematic methotrexate.^3^, ^11^ Whereas topical corticosteroids, heparin, high‐dose penicillin, and photoprotection‐reported no improvement. Wongkietkachorn K et al. treated with calcipotriol (synthetic 1,25‐dihydroxy vitamin D3) twice daily; an improvement was obtained only during the early treatment stage.^3^ Our patient was treated with colchicine tablets 0.5 mg per day, calcipotriol cream, and vitamin E tablets for 3 months. Partial pigmentation improvement was noted.

**TABLE 1:** Comparison of cases of LAM in the literature since 2013: patient demographics, location, characteristic clinical features, treatment, and response.

Cases (first author, year)	Sex\age (years)	Location	Characteristic clinical features	Treatment	Response
Patsatsi et al. (2013)^7^	Female\17	Trunk		Topical steroids and tacrolimus	No improvement
Villani et al. (2013)^8^	Male\8	Leg		No treatment	Spontaneous improvement
	Male\6	Trunk\Leg		Topical steroids	No improvement
	Female\9	Trunk		Non‐reported	
	Male\20	Trunk\Arm		Non‐reported	
Wongkietkachorn et al. (2013)^3^	Male\15	Trunk\Leg		Topical calcipotriol	Clinical improvement
Yüzcel et al. (2013)^9^	Female\23	Leg	The simultaneous presence of spotty, segmental lentiginosis, and LAM was observed	Non‐reported	
Golian et al. (2014)^10^	Male\16	Arm\Leg\Trunk		Non‐reported	
Zaouak et al. (2014)^11^	Female\21	Arm\Leg\Trunk		Methotrexate 20 mg/week for 6 months	Improvement of skin pigmentation and atrophy
Ahearn et al. (2015)^12^	Female\40	Arm\Trunk	Clinical lesions exhibited a biphasic pattern, emerging during late adolescence and into adulthood	Non‐reported	
Zahedi et al. (2015)^5^	Male\10	Arm	Onset was preceded by discolouration resembling bruises	Topical steroids, vitamin D analogues, retinoids, and hydroquinone	No improvement
Lis‐Świety et al. (2016)^13^	Female\26	Arm\Leg\Trunk	The condition manifested bilaterally and showed partial symmetry, with involvement noted on the chin	Non‐reported	
Yan et al. (2016)^14^	Male\28	Arm\Trunk	Involvement extended to the neck, with positive findings for ANA, ribonucleoprotein, and anti‐SM, along with decreased levels of IgM, CD4, and CD4/CD8	Topical tacrolimus for 4 months	No improvement
Tan and Tay (2016)^15^	Female\22	Arm\Leg\Trunk		Betamethasone, 0.1% cream twice a day for 6 months‐ hydroquinone 4% cream for 2 years	No improvement
Darung et al. (2017)^16^	Female\16		Expansion was observed on the left side of the chin, displaying a wrinkled surface	Topical tretinoin (0.05%) cream once at night	No data
Kharkar et al. (2018)^17^	Male\17	Trunk		Intralesional platelet‐rich plasma injections	Partial improvement in terms of reduction in the depth of the plaque. However, the texture and the colour remained the same
Zhang et al. (2020)^6^	Female\15	Arm\Trunk		Topical halometasone 0.5% cream and hydroquinone 2% cream for 2 months	No improvement
Wang and Zeng (2020)^18^	Female\ adolescent	Arm\Leg\Trunk		None	
Zhang et al. (2022)^4^	Female\24	Arm	Ultrasonic findings revealed a reduction in dermal thickness accompanied by increased echogenicity, along with decreased subcutaneous tissue exhibiting normal echogenicity	Non‐reported	
	Female \23	Trunk	The condition extended to the neck area, with ultrasonic characteristics indicating a decrease in dermal thickness but normal echogenicity, alongside normal subcutaneous thickness and echogenicity	Non‐reported	
	Female \23	Arm\Trunk	Ultrasonography displayed a diminished dermal layer with normal echogenicity, coupled with normal subcutaneous thickness and echogenicity	Non‐reported	
	Female \15	Arm	Ultrasonographic examination revealed reduced dermal thickness with normal echogenicity and normal subcutaneous tissue thickness with typical echogenicity	Non‐reported	
Tang and Wang (2023)^19^	Female \29	Leg\Trunk	The presence of positive antinuclear antibody and elevated levels of immunoglobulin M were noted	Oral glucoside tablets and topical glucoside ointment	No improvement
Our case (2023)	Female \18	Leg\Trunk		Calcipotriol cream 0,0055, colchicine tablets 0.5 mg per day, and vitamin E tablets for 3 months	Partial improvement in pigmentation

Abbreviations: LAM, linear atrophoderma of Moulin.

## 4 CONCLUSION

In conclusion, our case underscores the clinical diversity of LAM, exemplified by the atypical Blaschkoid distribution observed. This case prompts consideration of a potential novel variant within the spectrum of LAM, emphasising the necessity for comprehensive classification and management approaches. Further research is essential to elucidate the underlying mechanisms and clinical significance of these variants, ultimately improving diagnostic accuracy and therapeutic outcomes.

## CONFLICT OF INTEREST STATEMENT

None to declare.

## AUTHOR CONTRIBUTIONS

**Moatasem Hussein Al‐janabi**: Conceptualization (equal); data curation (equal); formal analysis (equal); project administration (equal); validation (equal); writing – original draft (equal); writing – review & editing (equal). **Sdrah Diab**: Data curation (equal); formal analysis (equal); investigation (equal); methodology (equal); resources (equal); validation (equal); visualization (equal); writing – original draft (equal). **Ghina Aljammal**: Data curation (equal); formal analysis (equal); investigation (equal); writing – original draft (equal). **Lina Kassab**: Data curation (equal); formal analysis (equal); writing – original draft (equal). **Zuheir Al‐Shehabi**: Supervision (equal); validation (equal); visualization (equal). **Lina Al‐Soiufi**: Resources (equal); supervision (equal); validation (equal); visualization (equal).

## ETHICS STATEMENT

Not applicable.

## PATIENT CONSENT

Written patient consent for publication was obtained.

## Data Availability

Research data are not found.
